# The Brain in (Willed) Action: A Meta-Analytical Comparison of Imaging Studies on Motor Intentionality and Sense of Agency

**DOI:** 10.3389/fpsyg.2019.00804

**Published:** 2019-04-12

**Authors:** Silvia Seghezzi, Eleonora Zirone, Eraldo Paulesu, Laura Zapparoli

**Affiliations:** ^1^Department of Psychology and NeuroMI – Milan Centre for Neuroscience, University of Milan-Bicocca, Milan, Italy; ^2^Ph.D. Program in Neuroscience, School of Medicine and Surgery, University of Milan-Bicocca, Milan, Italy; ^3^fMRI Unit, IRCCS Orthopedic Institute Galeazzi, Milan, Italy

**Keywords:** sense of agency, motor intention, action awareness, fMRI, PET, meta-analysis

## Abstract

Voluntary actions can be fractionated in different phenomena: from the emergence of intentions and the ensuing motor plans and actions, to the anticipation and monitoring of their outcomes, to the appreciation of their congruency with intentions and to the eventual emergence of a sense of agency. It follows that motor intention and the sense of agency should occur at different stages in the normal generation of willed actions. Both these processes have been associated with a fronto-parietal motor network, but no study has investigated to what extent the two experiences can be dissociated for the brain regions involved. To this end, we assessed the PET/fMRI literature on agency and intentionality using a meta-analytic technique based on a hierarchical clustering algorithm. Beside a shared brain network involving the meso-frontal and prefrontal regions, the middle insula and subcortical structures, we found that motor intention and the sense of agency are functionally underpinned by separable sets of brain regions: an “intentionality network,” involving the rostral area of the mesial frontal cortex (middle cingulum and pre-supplementary motor area), the anterior insula and the parietal lobules, and a “self-agency network,” which involves the posterior areas of the mesial frontal cortex (the SMA proper), the posterior insula, the occipital lobe and the cerebellum. We were then able to confirm this functional organization by a subsequent seed-based fMRI resting-state functional connectivity analysis, with seeds derived from the intentionality/sense of agency specific clusters of the medial wall of the frontal lobe. Our results suggest the existence of a rostro-caudal gradient within the mesial frontal cortex, with the more anterior regions linked to the concept of motor intentionality and the brain areas located more posteriorly associated with the direct monitoring between the action and its outcome. This suggestion is reinforced by the association between the sense of agency and the activation of the occipital lobes, to suggest a direct comparison between the movement and its external (e.g., visual) consequences. The shared network may be important for the integration of intentionality and agency in a coherent appreciation of self-generated actions.

## Introduction

Volitional or voluntary actions are crucial components of our daily life and they could be defined as “self-initiated” or “self-generated” actions (Passingham et al., 2010). Although much of the functioning of the voluntary motor systems occurs without the need of any conscious thought, humans are aware that they intend to move and are responsible for the consequences of their own acts. Our intentions to produce a movement (the so-called “motor intention” or volition) and the feeling of controlling our own motor acts and, through them, the events in the external world (the so-called “sense of agency”), represent two crucial components of the action awareness for voluntary acts.

We first describe in detail the concepts of motor intention and sense of agency, how they have been experimentally manipulated and their corresponding brain correlates as they would emerge from a traditional review of the literature. We then address two cognitive models that contextualized motor intention and sense of agency into different conceptual frameworks, from which distinct predictions about their neurofunctional underpinnings could be drawn. Finally, we introduce a formal meta-analysis of neuroimaging studies, that specifically tested these neurofunctional predictions and the validity of functional anatomical assignations in terms of their replicability across studies and dissociability along the intentionality/agency axes.

### The Conscious Motor Intention

Motor intentionality refers to the reasons that bring a subject to produce a specific action: indeed, intentional actions (e.g., actions that are caused by conscious intentionality) are defined as goal-directed, not externally triggered, not habitual and not automatic (Haggard, 2008).

Thus, they have been conceptualized as a form of motor decision making, free from the external constraints defined by sensory cues (Haggard, 2008). According to this approach, the dominant experimental setting for studying motor intention requires subjects to decide between different action alternatives in three main ways: each participant can be asked to choose which action to perform among a set of alternatives (Frith et al., 1991), or to execute a specific action choosing the timing of the movement (Lau et al., 2004a), by taking advantage of the Libet’s paradigm (Libet et al., 1983), or to decide whether to perform or to inhibit an action (Brass and Haggard, 2007). Functional imaging studies of motor intention have frequently shown activations in the anterior portion of the pre-SMA (Lau et al., 2004a), in the anterior cingulate cortex (ACC) (Zapparoli et al., 2017, 2018), in the SMA proper (Jenkins et al., 2000; Hoffstaedter et al., 2013), in the superior (Forstmann et al., 2006) and inferior (Wisniewski et al., 2016) parietal lobules, in the insular cortex (Krieghoff et al., 2009) and in the cerebellum (Lau et al., 2004b).

### The Sense of Agency

The sense of agency is another central aspect of action awareness and refers to the ability to recognize that an external sensory event has been caused by our behavior (self-agency) rather than by an external agent (external-agency).

Synofzik et al. (2008b) distinguished between two distinct forms of agency experience: the “judgment of agency” and the “feeling of agency.” The “judgment of agency” refers to the conceptual, interpretative, explicit judgments of being the agent of an outcome (“Did I do that?”). The “feeling of agency” represents the non-conceptual, implicit feeling of control that accompanies their own actions, in the absence of any conscious thought.

The dominant experimental paradigm addressing the “judgment of agency” involves the request of performing explicit judgments about whether a sensory event has been caused by one’s action (self-agency) or by another agent (external-agency). Typically, participants perform hand movements, see video feedbacks showing the target motor act and judge whether they are viewing their own action or not, basing either on spatial (Daprati et al., 2007) or temporal (Farrer et al., 2008) features of the seen movements, which could be experimentally manipulated. However, although explicit judgments of agency may be crucial in social contexts in which the attribution of the agency has important consequences in the domain of responsibility, our everyday experience of agency does not generally involve explicit judgments (Haggard, 2017). For this reason, the type of experience that can be captured by an overt rating can differ considerably from our ordinary sense of agency. Accordingly, the “feeling of agency” has been investigated by means of implicit paradigms, which are able to capture this feeling without requiring people to overtly think about their agency. For example, in experiments taking advantage of the “intentional binding” effect, participants are asked to report the perceived time either of the action or of a subsequent sensory event (Haggard et al., 2002, for a critical review see also Moore and Obhi, 2012). Alternatively, the perceptual attenuation of the self-generated action consequences has been used as an implicit measure of the feeling of agency. This effect, defined as “sensory attenuation” (Frith et al., 2000; Blakemore et al., 2002), refers to the subjective perception of a self-generated sensory stimulus as less intense than an identical externally generated stimulus.

Neuroimaging studies, mainly based on explicit agency judgments, have shown the association between the self-agency and the activity of the pre-SMA (Tsakiris et al., 2010), the SMA proper (Farrer and Frith, 2002; Kühn et al., 2013), the superior (Matsuzawa et al., 2005) and inferior parietal lobules (Matsuzawa et al., 2005; Renes et al., 2015), the insular cortex (Farrer and Frith, 2002) and the cerebellum (Schnell et al., 2007; Fukushima et al., 2013).

At the nominal level, the comparison between these neuroimaging findings and those concerning motor intentionality reveal a great degree of overlap. The remaining question on whether these could be dissociated on a finer anatomical grain represents the issue that we will address in the empirical part of this manuscript.

### Models of Conscious Experience of Voluntary Action

The conscious experience of motor intention and agency have been contextualized in different theoretical frameworks. It is still unclear whether motor intention and agency might be underpinned by a shared general-domain reconstructive process (Wegner and Wheatley, 1999) or by two partially-independent cognitive processes or stages of action awareness (Frith et al., 2000; Wolpert and Ghahramani, 2000; Haggard, 2005). According to the first framework, the two phenomena would result from a unique cognitive process of *post hoc* reconstructive attribution (Wegner and Wheatley, 1999). The second framework suggests that they might rely on different constructive processes based on the generation of specific internal models and predictions (Frith et al., 2000; Wolpert and Ghahramani, 2000; Haggard, 2005).

#### The Reconstruction of Action Awareness: The Theory of Apparent Mental Causation

The “theory of apparent mental causation” posits a reconstructive view of the conscious experience of voluntary action (Wegner and Wheatley, 1999). Wegner (2003) conceptualizes the conscious experience of acting as a *post ho*c inference, occurring after the end of the movements (Wegner, 2003). The inference occurs in accordance with three main principles: priority, consistency, exclusivity. If (1) a thought becomes conscious just before an action (*priority*), (2) the thought is consistent with the action (*consistency*) and (3) it is not accompanied by alternative apparent causes of the action (*exclusivity*), we experience conscious intention and ascribe authorship to ourselves for the action. We do not have access to any direct signal about the true intention to perform an action and we do not use it when the sense of agency is generated (Wegner, 2003). To use the words of Patrick Haggard reviewing this model, “*if I first feel my finger moving*, *and then hear an auditory tone*, *I will infer a conscious intention to move*, *and reconstruct the intention as being the cause of both movement and the tone*” (2003). Accordingly, Wegner’s view suggests that the mind can produce both the tricks that “I” have caused external events and “I” had a preceding intention to make an action (Haggard, 2005). Therefore, intention and agency experiences could be both seen as a result of a shared *post ho*c reconstruction occurring after the end of the movement; they could both be considered as an “illusion of mental causation” retrospectively inferred to explain behavior, rather than a direct report of the pre-motor brain activity (Wegner, 2003). This theory has received some support from studies showing the easily-biased nature of action awareness in situations of ambiguous authorship (Wegner and Wheatley, 1999; Aarts et al., 2005). For example, an experiment by Wegner and Wheatley (1999) demonstrates that particular environmental circumstances can lead subjects to erroneously perceive the intention of performing actions that are actually not caused by them. In that study, both subjects and external agents could be plausible causes of given action outcomes. Within such context, an acoustically presented prime encouraged subjects to retrospectively attribute to themselves conscious intentions of actions that were performed by another person.

#### The Construction of Action Awareness: The Comparator Model

Action awareness can also be explained in the framework of computational models of movement control, such as the comparator model (Frith, 1987; Wolpert, 1997; Wolpert and Ghahramani, 2000).

Central to the comparator model is the idea that the motor control system makes use of internal models which internally represent the motor-to-sensory transformations and how these are implemented in the physical world. There are two main types of internal models underpinning motor control: inverse and forward models. The control of action mainly depends on the coupling of these internal models through a series of comparators, namely mechanisms that compare signals and use the result of the comparison for the regulation of the system. Accordingly, an action starts with an intention or a desired goal state. An inverse model computes the appropriate motor commands that are required to achieve the desired goal from the current state of the system and the environment. A forward model represents the causal flow of the ongoing motor process and uses the so-called “efference copy” of the current motor program to make predictions of the sensory consequences of the ongoing movement. These predictions are then compared with the real outcomes of the actions (Haggard, 2017). This mechanism can be used to (1) adjust the current motor command in order to rapidly correct the movements, to (2) attribute the agency for the produced action outcomes, to (3) cancel out or attenuate sensory feedbacks that are self-produced, the phenomenon known as “sensory attenuation” (Frith et al., 2000; Blakemore et al., 2002). In particular, if predicted and estimated actual state are congruent, the sensory event is attributed to one’s own agency, while a mismatch prevents the consequence to be attributed to one’s own action (Blakemore and Frith, 2003). For what concerns the sensory attenuation phenomenon, such effect is supposed to occur when the predictions of the outcome of the action make reafferent perception of the actual effect redundant (Haggard, 2017).

In a nutshell, two crucial arguments are particularly useful for our attempt to characterize action awareness. First, the comparator model suggests a strict temporal structure in action awareness: motor intention precedes the preparation of the motor act, which necessarily precedes the movement of the body. The establishment of a link between our motor intentions and our actions and between our actions and their external outcomes determines the sense of agency (Haggard and Clark, 2003). Second, the comparator model, by directly connecting action awareness to action perception and motor processes, could be a parsimonious explanation of the conscious experience of action, since it is able to explain it as an intrinsic property of action processing (Synofzik et al., 2008a).

#### The Neural Predictions of the Models

Although the authors of the two aforementioned models did not overtly propose specific neurofunctional predictions, the two frameworks allow us to hypothesize different sets of neuroanatomical patterns associated with motor intention and agency.

According to the reconstructive view, action awareness may arise from an inferential ‘sense-making’ process, which uses sensory evidence about physical events in order to reconstruct the intention and the agency of the action after its execution. As a consequence, as Pacherie (2008) hypothesized in her work on the phenomenology of action, the theory of apparent mental causation implies that the process of action awareness generation should be considered as separated from the process involved in action specification and control. Conversely, this might result from the same mental processes typically involved in a general appreciation of causality, which process environmental and contextual cues to infer causality (Wegner and Wheatley, 1999).

Consequently, the following neuroanatomical predictions might be hypothesized: first, the existence of a shared neural network implied in the formation of the feelings of intentionality and agency; second, the involvement of non-motoric neural systems in the retrospective reconstruction of causality.

Alternatively, action awareness might depend on a constructive process, depending on consecutive analytical steps taking advantage of efferent motor command signals. As a consequence, the comparator model allows one to hypothesize a different functional anatomical scenario whereby the translation of general goals into specific motor intentions and the occurrence of the sense of agency should be associated with partially-distinct brain network, deeply embedded in the sensory-motor system. Two additional predictions complement this argument. First, one should expect a reduced activity associated with the self-produced movements in the sensory cortices, according to the sensory attenuation hypothesis. Second, the comparator model necessarily needs to consider the problem of how “motor intention” and “agency” specific networks interact. One possibility is that this might occur by means of effective connectivity through separate systems. Alternatively, one could predict a shared set of brain regions at the interface between the intention and the agency-specific processes. This latter possibility is suggested by the assumption that the sense of agency should partly depend on the experience of intentionality itself (Haggard, 2017).

Anatomical considerations on the nature of the areas that may prove to be significantly involved in intention and agency experiences may ease the interpretations of anatomical findings and their explanatory power in favor of the aforementioned theories: for example, a systematic and exclusive involvement in action awareness of regions connected with the spinal cord may pull the argument in favor of a motoric interpretation whereby the motor signals explicitly contribute to the construction of such mental state, a position much closer to the comparator hypothesis. The impossibility of separating “intention specific” brain areas form those implicated with agency would rather militate in favor of a cognitive reappraisal theory – *post hoc* – reconstruction of the consequences of actions as self-generated.

### Aim of the Study

To date, the two cognitive constructs of motor intention and sense of agency have never been manipulated together in the same experimental context with neuroimaging measures, mainly due to the lack -at present- of neuroimaging techniques with sufficient spatio-temporal resolution to capture the neurofunctional correlates of so closely interwoven constructs. One other possibility is to rely on a quantitative assessment of the results of specific experiments in which intentionality or agency have been studied separately using a strict cognitive subtraction approach.

To this end, we adopted a meta-analytical approach that allowed us to formally compare, with statistical measures, the neural correlates of motor intention and sense of agency, their overlap and dissociations.

We reasoned that the assessment of the degree of overlap and dissociations of the neural correlates of the two constructs and the nature of the recruited networks, would have proved useful in disentangling the anatomical foundations of motor intention and the experience of agency and tell whether we should favor a unitary retrospective mechanism (Wegner’s view), or a mechanism more akin to those postulated by the comparator model.

## Materials, Methods, and Results

In this paper, we adopted a two-level analysis strategy. We first performed an explorative meta-analysis of the existing neuroimaging literature on action awareness by means of a hierarchical clustering (HC) algorithm, in order to provide a quantitative review of the neuroimaging studies on motor intention and sense of self-agency. To this aim, we identified the eligible studies, based on the degree of overlap with our research question. We then extracted and classified the coordinates according to the type of contrasts defined in each study. We finally applied a HC algorithm to the dataset, followed by statistical inferences on the resulting clusters.

We employed the HC procedure in order to overcome the disproportion of peaks associated with the two levels of the factor of interest within the source dataset. In fact, the HC method has the advantage of allowing the statistical exploration of each resulting cluster (cluster composition analysis) by comparing the proportion of the foci belonging to either level of the factor of interest within each cluster with the overall distribution of foci in the whole dataset. By applying this prior likelihood estimate of the expected number of foci in any given cluster under the null hypothesis, given the overall proportions of construct specific foci, we were able to efficiently handle the greater number of foci associated with the motor intention level compared with the sense of self-agency one and still perform our inferences (see below for the details).

Two subsequent analyses complemented this explorative meta-analysis: a conjunction analysis between the motor intention and the sense of agency data set, and a cluster analysis focused on the sense of external-agency, in order to test the sensory attenuation hypothesis formulated by the comparator model of action.

Finally we implemented a confirmative analysis, where the main results of the HC analysis were employed as ROIs for a resting-state functional-connectivity (rsFC) analysis of fMRI data, in order to further characterize the functional networks involved in action awareness and to add some face validity to our anatomical inferences based on a totally independent data set. Functional connectivity refers to the functionally integrated relationship between spatially separated brain regions. Indeed, whereas our meta-analytical approach allows us to describe different sets of brain regions selectively associated to the performance of “motor intention task” or “agency task,” the rsFC analysis allows a deeper and task-free functional characterization of such brain networks. These procedures are described in detail below.

### Explorative Meta-Analysis

#### Methods

##### Data collection and preparation

First, we interrogated the PubMed database^1^ in April 2018^2^ with the following keywords: “Motor intention and fMRI,” “Motor intention and PET,” “Motor intention and neuroimaging” and “Sense of agency and fMRI,” “Sense of Agency and PET,” “Sense of agency and neuroimaging” for the two datasets, respectively.

Second, we performed a detailed inspection of the resulting manuscripts and we excluded the studies that did not report data by using stereotactic coordinates (either MNI – Montreal Neurological Institute – or Talairach atlases) and did not recruit only healthy adult subjects.

The final dataset included 17 studies that investigated the functional correlates of motor intention and 14 studies that explored the sense of self-agency (see Supplementary Table S1), for a total of 342 peaks of activation. The studies addressing motor intention have mostly employed neuroimaging procedures similar to the “Free selection paradigm” (Lau et al., 2004b), in which two experimental conditions are compared: a condition in which responses are externally determined by a cue and a condition in which the participants have to choose freely between different motor responses. On the other hand, the brain correlates of the sense of agency have been mainly investigated by manipulating the visual feedback of the movement. In order to functionally characterize each cluster by means of a subsequent cluster composition analysis, each peak within the dataset was classified according to the two levels of the only factor of interest (action awareness): motor intention and sense of self-agency. In particular, 246 peaks were associated with motor intention and 96 peaks were associated with the sense of self-agency. We employed the activation foci resulting from simple comparison between the factor of interest and the control condition [e.g., intentional action > rest condition; intentional action > stimulus-driven action; self-agency (visuo-motor congruency) > rest; self-agency (visuo-motor congruency) > external-agency (visuo-motor discrepancy)] and parametric regressions (e.g., parametric function of the BOLD response as a function of visuo-motor congruency degree). For a more detailed contrast characterization see the Supplementary Table S1. Further, all the Talairach coordinates were converted to MNI space through the Talairach to MNI (SPM) transformation implemented in the software CluB (Clustering the Brain, see below).

The final dataset included 474 participants (mean age = 27.35 ± 4) and 34 contrasts.

##### Cluster analysis and cluster composition analysis: motor intention and sense of self-agency

To identify anatomically coherent regional effects, we first performed a cluster analysis using the unique-solution clustering algorithm developed by Cattinelli et al. (2013). This method, implemented in a suite of MATLAB (2014a MathWorks) and C++ scripts called “CluB” (the CluB software can be found here^3^), takes into account the squared Euclidian distance between each couple of foci included in the dataset; the clusters with minimal dissimilarity are then recursively merged by means of the Ward’s criterion (Ward, 1963), with the aim of minimizing the intra-cluster variability and maximizing the between-cluster variability (Cattinelli et al., 2013).

The spatial resolution of our analyses was set to be of 5 mm, corresponding to the maximum mean spatial variance within each cluster in the three directions. The output of the cluster analysis was then employed to perform the subsequent cluster composition analysis. This procedure allows a non-parametric *post hoc* exploration of the composition of each cluster, providing a statistical account of the degree of association of each cluster with the levels of the factor of interest. In particular, after extracting the proportion of peaks associated with the factors of interest within the whole dataset, the software computes the proportion of activation peaks belonging to either factor within each cluster. Then, the proportions observed within each cluster are compared to the overall prior likelihood with a binomial test, under the null hypothesis that the proportion of foci within each cluster is different from the prior likelihood.

After the clustering procedure, the centroid coordinates of each resulting cluster were labeled according to the Automatic Anatomic Labeling (AAL) and then manually checked by visual inspection using the MRIcron^4^ (Rorden and Brett, 2000) software.

##### Activation likelihood estimation conjunction analysis: motor intention and sense of agency

The HC procedure does not allow a statistical characterization of the conjunction between the clustering results, in order to identify the voxels commonly associated with both the two levels of interest. This limit has been circumvented by employing the ALE conjunction analysis provided by the Ginger-ALE software (Eickhoff et al., 2009; Turkeltaub et al., 2012), that creates a conjunction image using the voxel-wise minimum value of the input ALE images (see for example, Murray et al., 2012).

We first conducted two separate ALE analyses, one focused on motor intention and one on the sense of self-agency. We employed the Turkeltaub Non-Additive method (Turkeltaub et al., 2012) with the general statistical threshold set to *p* < 0.05 (uncorrected), resulting in an ALE map and in a corresponding cluster report. Conjunction analysis was then carried out to determine the intersection between the meta-analyses on intentionality and agency. We employed a statistical threshold of *p* < 0.05 FDR (pID) corrected for multiple comparisons and a cluster-size threshold of 300 mm^3^, such that only clusters of contiguous voxels exceeding a volume of 300 mm^3^ were considered. All the ALE meta-analyses were conducted in the standard MNI space (Eickhoff et al., 2009; Turkeltaub et al., 2012). The maps of the ALE values were overlaid on a ch2better.nii.gz template using MRIcron software (Rorden and Brett, 2000).

##### Cluster analysis: external-agency (sensory attenuation hypothesis)

To test the hypothesis that self-generated actions (self-agency) are associated with reduced activations at the level of the sensory cortices with respect to externally-generated movements (external-agency), we created a separate database listing only the activation foci that resulted from the following contrasts: external-agency > rest, external-agency > self-agency. The final dataset included 11 studies that investigated the sense of external-agency, with 142 peaks of activation (see Supplementary Table S2). No similar analysis was possible for the fMRI signal reductions in the intentionality studies as the data available were a mere 53 peaks from 8 studies.

The cluster analysis procedure, with one factor characterized by only one level, does not allow to make any statistical inference on the clustering results. This limit has been previously circumvented by combining the HC approach with the ALE procedure (Paulesu et al., 2014) provided by the Ginger-ALE software (Eickhoff et al., 2009; Turkeltaub et al., 2012).

For the spatial cross-validation ALE we employed the Turkeltaub Non-Additive method (Turkeltaub et al., 2012), with the general statistical threshold set to *p* < 0.05 FDR (pID); the resulting maps were overlapped with the cluster analysis map with the “intersection” function in the software MRIcron (Rorden and Brett, 2000). Only the clusters that fell in this intersection map were further discussed. The overlapping clusters have been marked with an asterisk in Table 3.

#### Results

##### Cluster analysis and cluster composition analysis: motor intention and sense of self-agency

The cluster analysis yielded 68 clusters (CL), containing on average 5 foci (range: 1–17 peaks). The mean standard deviation along the three axes was 4.44 mm (*x*-axis), 4.70 mm (*y*-axis), and 4.95 mm (*z*-axis). See Supplementary Table S3.

**FIGURE 1:**
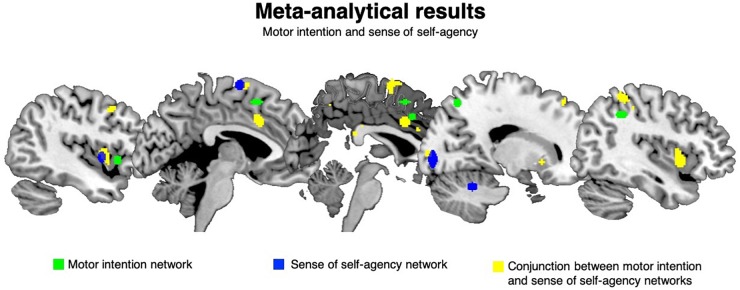
Meta-analytical results: motor intention and sense of self-agency. The green clusters are significantly associated with motor intention, the blue clusters are significantly linked to the sense of self-agency while the yellow clusters are the ones shared by motor intention and the sense of self-agency.

The clusters resulting from the HC procedure were then submitted to the cluster composition analysis to test the degree of association between each cluster and each level of the factor of interest, by means of a binomial test. The results revealed that five clusters were significantly associated with motor intention and four with the sense of self-agency. See Table 1 and Figure 1.

**Table 1:** Results of the cluster composition analysis: motor intention vs. sense of self-agency.

Brain regions (BA)	MNI coordinates																	
	Left hemisphere									Right hemisphere								
	Cluster ID	*k*	MNI coordinates			Standard deviation			*P*-value	Cluster ID	*k*	MNI coordinates			Standard deviation			*P*-value
			*x*	*y*	*z*	*x*	*y*	*z*				*x*	*y*	*z*	*x*	*y*	*z*	
**(a) Intentionality**		
Middle cingulum (24)										53	17	1	19	39	4.6	4	3.4	0.03
Pre-supplementary motor area (6)	58	10	–**3**	**12**	**53**	7.1	6.5	2.5	0.04									
Anterior insula	41	9	–41	17	–2	4.9	4.4	5	0.05									
Superior parietal lobule (7)										45	9	18	–67	52	5.4	3.6	4.1	0.05
Inferior parietal lobule (40)										22	13	39	–45	41	4.4	5.3	3.8	0.05
**(b) Sense of agency**		
Supplementary motor area (6)	24	5	–**7**	–**4**	**69**	4.7	5	5	0.02									
Posterior insula	28	7	–41	2	1	4.4	4.1	5.9	0.02									
Calcarine scissure (18)										59	5	18	–90	–1	6	4.2	8.4	0.02
Cerebellum										67	5	24	–53	–27	8.8	7.8	6.7	0.02


*For each cluster, we report: the cluster ID; the number of foci falling within the cluster; the centroid coordinates in the MNI stereotaxic space and the standard deviation (sd) of the Euclidean distance from the centroid along the three axes; the p-values associated with the binomial test. We reported in BOLD the coordinates used as seeds for the rsFC analysis.*

Motor intention specific clusters were located in the right inferior parietal lobule (CL22), in the left anterior insula (CL41), in the right superior parietal lobule (CL45), in the right middle cingulum (CL53) and in the left pre-SMA (CL58). See Table 1a and Figure 1 (areas in green).

Self-agency specific clusters were found in the left SMA (CL24), in the left posterior insula (CL28), in the right calcarine fissure (CL59) and in the right cerebellum (CL67). See Table 1b and Figure 1 (areas in blue).

##### Activation likelihood estimation conjunction analysis: motor intention and sense of self-agency

The conjunction analysis returned 14 clusters, with a mean extension of 766.3 mm^3^ and an ALE score of 0.005–0.009. In particular, the bilateral middle insula (CL1 and CL2), the left (CL3 and 10) and right (CL14) putamen, the left precentral gyrus (CL4), the left middle cingulum (CL5), the right SMA proper (CL6), the left middle frontal gyrus (CL7), the right superior parietal lobule (CL8), the right anterior cingulum (CL9), the left thalamus (CL11), the left superior frontal gyrus (CL12) and the right calcarine scissure (CL13) represented a network shared by motor intention and the sense of self-agency. See Table 2 and Figure 1 (areas in yellow).

**Table 2:** Results of the ALE conjunction analysis: motor intention and sense of self-agency.

Cluster #	Brain regions (BA)	Volume (mmˆ3)	Extrema value	MNI coordinates of the Weighted Center					
				Left hemisphere			Right hemisphere		
				*x*	*y*	*z*	*x*	*y*	*z*
1	Middle insula	2048	0.008536732				44.3	9.2	–1.6
2	Middle insula	1200	0.008267164	–42.2	6.2	1			
3	Putamen	1096	0.008439786	–26.8	–0.7	–0.9			
4	Precentral gyrus (6)	792	0.005983784	–34.6	–12.9	60.5			
5	Middle cingulum (24)	720	0.007679645	–2.9	13	34.6			
6	Supplementary motor area (6)	704	0.006421017				0.7	1.7	68.3
7	Middle frontal gyrus (46)	680	0.007679653	–35.6	44.6	29.1			
8	Superior parietal lobule (40)	680	0.008001605				41.6	–44.1	58
9	Anterior cingulum (24)	640	0.007679645				5.8	25	27.7
10	Putamen	536	0.007680166	–13.9	8.4	–6.8			
11	Thalamus	520	0.006345872	–10.4	–17.9	11			
12	Superior frontal gyrus (6)	480	0.006096084	–24.1	–2.4	65			
13	Calcarine scissure (18)	320	0.006865089				13.7	–94.4	4
14	Putamen	312	0.005142419				13.1	11	–2.8


*For each cluster, we report: the cluster ID; the volume (mm^*3*^), the ALE score and the centroid coordinates in the MNI stereotaxic space.*

It is worth noting that these areas of shared local effects overlap with some of the clusters identified with CluB where the value of the binomial test was very far from significance in any direction, to testify a similar proportion of peaks from motor intention and the self-agency datasets. See Supplementary Table S3.

The advantage of the ALE approach here was to allow an inference about significance to the level of overlap of the separate regional effects.

##### Cluster analysis: external-agency (sensory attenuation hypothesis)

The cluster analysis yielded three clusters, containing on average 7.7 foci (range: 7–9 peaks). The mean standard deviation along the three axes was 4.3 mm (*x*-axis), 5.0 mm (*y*-axis), and 4.9 mm (*z*-axis).

**FIGURE 2:**
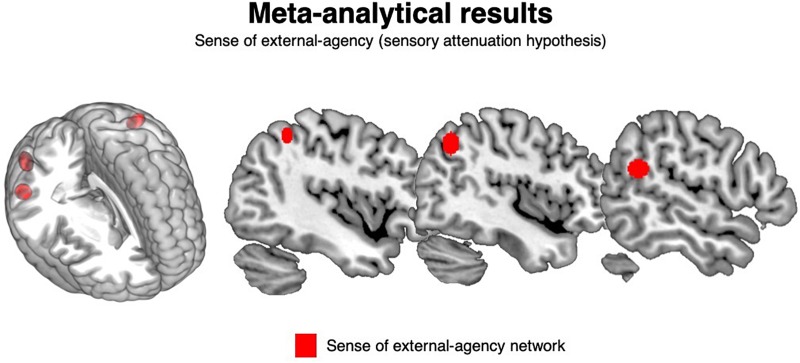
Meta-analysis results: external-agency. The clusters significantly associated with the sense of external-agency are depicted in red.

These clusters were located in the left inferior parietal lobule, in the right superior temporal gyrus and in the right angular gyrus. See Table 3 and Figure 2.

**Table 3:** Results of the cluster analysis: external agency.

Brain regions (BA)	MNI coordinates															
	Left hemisphere								Right hemisphere							
	Cluster ID	*k*	MNI coordinates			Standard deviation			Cluster ID	*k*	MNI coordinates			Standard deviation		
			*x*	*y*	*z*	*x*	*y*	*z*			*x*	*y*	*z*	*x*	*y*	*z*
Inferior parietal lobule (40)^∗^	37	7	–46	–48	51	7.7	3.9	6.3								
Superior temporal gyrus (22)									6	7	45	–60	43	4.5	6.6	9.0
Angular gyrus (39)									33	9	54	–49	22	5.9	6.2	5.5


*For each cluster, we report: the cluster ID; the number of foci falling within the cluster; the centroid coordinates in the MNI stereotaxic space and the standard deviation (sd) of the Euclidean distance from the centroid along the three axes. ^∗^Clusters overlapping with the ALE cross-validation analysis.*

### Confirmative Analysis: Functional Connectivity Data

#### Motivation

The results of the HC and ALE analyses are very encouraging in indicating that the “motor intention” and the “agency” networks can be teased apart one from the other, yet with some substantial overlap. If these functional anatomical patterns are somewhat hard-wired, one should be able to demonstrate such separations and convergence also on an independent data-set; this could be done taking advantage of fMRI resting-state functional data, using a seed-based rsFC analysis focused on the main clusters identified by the HC analysis. We focused this analysis on the pre-SMA and the SMA proper, as they have a crucial role in planning and initiating a voluntary action (Passingham and Lau, 2017; Zapparoli et al., 2018), in the consciousness of motor intention (Lau et al., 2004a) and in the explicit (Matsuzawa et al., 2005) and implicit (Kühn et al., 2013) experience of agency. Finally, our previous HC analysis suggested a rostro-caudal gradient within the mesial wall of the frontal lobe that we wanted to test some further.

#### Methods

##### Participants

Thirty-two subjects (mean age: 27.52 ± 5.7 years; mean education level 14.84 ± 3.2 years; 12 males and 20 females) without any cognitive, neurological, or psychiatric illness participated in the resting-state fMRI study. They were all right-handed as assessed by the Edinburgh handedness inventory (Oldfield, 1971). Each subject was asked to stay with their eyes closed, awake and, as far as possible, not to think about anything. The study protocol was approved by the Ethics Committee ASL Città di Milano, and informed written consent was obtained from all subjects.

##### fMRI data acquisition and analysis

MRI scanning was performed with a Siemens *Avanto* 1.5T scanner equipped with gradient-echo echo-planar imaging. Before the acquisition of functional data, high-resolution T1-weighted structural images were acquired (flip angle 35°, TE 5 ms, TR 21 ms, FOV 256 × 192 mm, matrix 256 × 256, TI 768, 160 slices with 1 mm × 1 mm × 1 mm voxels). Echo-planar imaging gradient-echo fMRI scans [flip angle 90°, echo time (TE) = 60 ms, TR = 3000 ms, field of view = 250 × 250 mm, and matrix = 64 × 64, slice thickness = 4 mm] were then acquired (212 volumes). The first 10 volumes of each sequence were discarded from the analyses.

##### Data analysis

Statistical Parametric Mapping (SPM) version 12 ^5^, in conjunction with Functional Connectivity (CONN) toolbox ^6^ software were used for data analysis.

The pre-processing of resting-state fMRI data was conducted according to the default pipeline included in CONN-fMRI Functional Connectivity toolbox (version 18 ^7^) including realignment and unwarping, slice-timing correction, outlier detection (Artifact Detection Tool, conservative settings: 95 percentiles in normative sample), structural segmentation and normalization, functional normalization, smoothing (10-mm Gaussian kernel) and band-pass filtering (0.008 < *f* < 0.09 Hz) to reduce the effect of low-frequency drift and high-frequency noise.

The CONN toolbox then used a component-based noise correction method (CompCor) to identify and remove the principal components of physiological and other sources of noises from white matter and cerebral spinal fluid. Additionally, the confounding effect of the movement-related parameters (six dimensions with their first order derivative) was removed.

In the first level analysis, the toolbox computes the Pearson’s correlation coefficients between the time course of the fMRI signal of the seed regions selected and the time course of each voxel in the brain separately to generate the parametric seed-to-voxel correlation map. A seed-to-voxel correlation map was computed for each subject for each seed. Correlation coefficients were then converted to *z*-scores using the Fischer’s transform to allow for subsequent GLM analysis.

Two analyses were made, one for each of the two seeds regions from the medial wall of the frontal lobe that were specifically associated with intentionality (pre-SMA) and agency (SMA proper).

Following the first level analysis, each seed-to-voxel correlation map (one for each subject) were imported from the CONN toolbox in SPM12 for further analysis.

In the second-level analysis, the connectivity maps of the pre-SMA/SMA from all participants were compared by means of a paired *t*-test.

The analyses identified regions that showed a difference in functional connectivity between seed/condition specific maps: the comparison pre-SMA_intention_ map > SMA_self-agency_ map identified areas of functional connectivity more related with the motor intention network; the comparison SMA_self-agency_ map > pre-SMA_intention_ map identified areas of functional connectivity more closely associated with the self-agency network.

Finally, as for the meta-analysis, to identify potentials areas of overlap in the connectivity maps, we also run a conjunction analysis between the simple effects of the connectivity maps based on the pre-SMA_intention_ specific cluster and those emerging for the SMA_self-agency_ specific cluster.

The regions described survived a canonical cluster-level FWE *p* < 0.05 correction (voxelwise uncorrected threshold: *p* < 0.001), in line with recent suggestions by Flandin and Friston (2017).

#### Results

**FIGURE 3:**
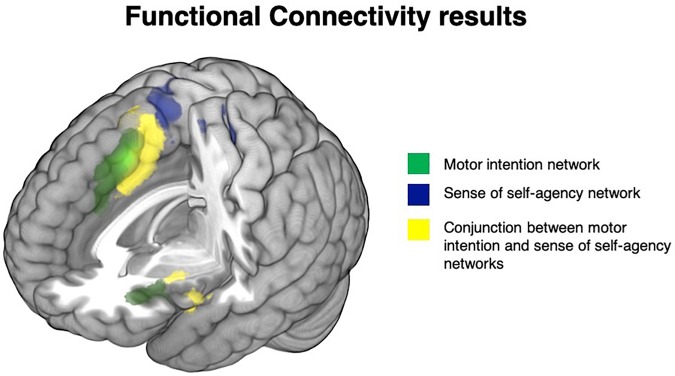
Functional connectivity results: brain regions specifically functionally connected with the pre-SMA (intentionality network) are depicted in green, the brain areas specifically functionally connected with the SMA (self-agency network) are depicted in blue, while the network shared by the pre-SMA (intentionality network) and the SMA (self-agency network) are depicted in yellow.

The seed located in the pre-SMA, associated with motor intention studies, was specifically connected with the anterior cingulum bilaterally, the left inferior frontal gyrus, the left precentral gyrus and the left anterior insula. See Figure 3, areas in green, and Supplementary Table S4A.

On the contrary, the portion of SMA significantly associated with the sense of agency (SMA proper) was connected with the paracentral and postcentral gyrus bilaterally, the left middle cingulum, the right precentral gyrus and the right superior parietal lobule. See Figure 3, areas in blue, and Supplementary Table S4B.

The conjunction analysis between the connectivity maps revealed a shared network involving the bilateral middle cingulum, the bilateral SMA, the bilateral anterior, middle and posterior insula, the bilateral superior temporal pole, the left inferior frontal gyrus pars opercularis, the left middle frontal gyrus, the left anterior cingulum, the left supramarginal gyrus and the left putamen. See Figure 3, areas in yellow, and Supplementary Table S4C: these illustrate the high level of congruency of the results generated by the meta-analyses and the seed-based functional connectivity maps on the independent resting state fMRI data.

## Discussion

The current study was conducted in order to deepen the investigation of the neurofunctional underpinnings of action awareness, by formally exploring, as quantitatively as possible, the available fMRI/PET literature. We focused our attention on the crucial components that define action awareness, motor intention and the sense of agency, and on two cognitive models that contextualize these two concepts in different frameworks.

Current theories suggest two possible processes at the basis of conscious awareness for voluntary acts: action awareness may arise from an inferential ‘sense-making’ process, which uses sensory evidence about physical events in order to reconstruct the intention and the authorship of the action after its execution (Wegner and Wheatley, 1999); alternatively, action awareness might depend on a constructive process, depending on efferent motor command signals (Wolpert and Ghahramani, 2000).

There are crucial differences between the reconstructive (Wegner and Wheatley, 1999) and the constructive (Wolpert and Ghahramani, 2000) views of action awareness: according to the reconstructive view, conscious experience of action is essentially a retrospective “illusion”; conversely, the constructive view postulates that it is an important aspect of sensorimotor neural activity, which can be experimentally approached as a motor phenomenon (Haggard and Clark, 2003).

The two models loosely imply certain non-equivalent functional anatomical predictions, and a meta-analysis has the advantage, at the very least, to make inferences about findings that repeat themselves, if they do, and to quantify the reliability of such repeats.

With this in mind, we can now discuss our findings also in the light of the reconstructive and constructive views described before.

### Common or Distinct Neural Substrates for Motor Intention and Sense of Agency?

#### HC Data

Our results show the existence of a common brain network shared by motor intention and the sense of self-agency, involving a diffuse neural system including premotor and prefrontal areas, such as the left superior and middle frontal gyri, the right anterior cingulum, the left middle cingulum, the right SMA, the left precentral gyrus, but also areas outside the frontal lobe at the level of the middle insula bilaterally, the right superior parietal lobule, the right calcarine scissure and in subcortical structures, such as the left thalamus and the bilateral putamen.

Beyond this shared network, we highlighted a segregation of the neural circuits associated with action awareness. In particular, our results suggest the existence of a rostro-caudal gradient in the mesial frontal cortex: the more anterior regions, such as the pre-SMA, associated with the experience of intentionality, a more posterior region of SMA resulted shared by motor intention and the sense of agency and, finally, the more posterior areas, such as the SMA proper, specifically related to the sense of agency. The association of the pre-SMA to the concept of intentionality is consistent with the shared area of activation of three intentional tasks reported in Zapparoli et al. (2018), a study that has been published after the conclusion of these analyses and not included here.

Furthermore, our results suggest the existence of a second rostro-caudal gradient in the brain areas supporting action awareness, located in the insular cortex. In particular, while the insular anterior region was significantly associated with the experience of intentionality, the middle insula was shared by the motor intention and sense of self-agency studies and the posterior insula was specifically linked to the feeling of self-agency.

The dissociation between intentionality and agency involved also brain regions well outside the median wall of the frontal lobe and the insular cortex, in a functionally-specific manner. For example, for motor intention the data clustered in the right middle cingulum and in the right parietal lobules. For what concerns the agency experience, our data clustered also in the right occipital lobe and in the right cerebellum.

#### Functional Connectivity Data

We then focused our analyses on the most interesting functional dissociation emerging from our data, located in the frontal midline: the dissociations between pre-SMA and SMA proper. We focused this analysis on such regions as they have a crucial role in planning and initiating a voluntary action (Passingham and Lau, 2017; Zapparoli et al., 2018), in the consciousness of motor intention (Lau et al., 2004a) and in the explicit (Matsuzawa et al., 2005) and implicit (Kühn et al., 2013) experience of agency. Finally, our previous HC analysis suggested a rostro-caudal gradient within the mesial wall of the frontal lobe that we wanted to test some further.

These clusters (centroids: pre-SMA MNI: -3, 12, 53, *sd*: 7.1, 6.5, 2.5; SMA MNI: -7, -4, 69, *sd*: 4.7, 5.0, 5.0) were used to compute seed-to-voxel connectivity maps in a sample of 32 healthy adult volunteers: this analysis can be regarded as complementary and confirmative with respect to the meta-analytical one. Indeed, whereas our meta-analytical analysis allows the quantitative description of the brain areas which have proven to be active during the performance of tasks differently associated with motor intention and the sense of self-agency, the functional connectivity analysis experimentally defines a set of regions whose activity correlates with one of the selected ROI. In other words, it allows the definition of the functional networks in which the ROIs are involved (Horwitz, 2003).

The results of this analysis showed a wide overlap with the HC data and further supported the dissociation between the neural network associated with intentionality and the network linked to the sense of agency. In particular, the pre-SMA associated with motor intention proved to be functionally connected with a set of frontal and prefrontal areas, such as the anterior cingulum and the inferior frontal areas and the anterior insula. Conversely, the SMA proper, specific for the sense of self-agency, was linked to the sensorimotor areas, such as the postcentral gyrus and the posterior insula.

Much as in the conjunction analysis performed in the meta-analysis section, the rsFC data confirmed also regions of intersection at the inner boundaries of the two specific networks.

Taken together, this evidence suggests that, outside a common network shared by both levels of action awareness, motor intention and the agency experiences are functionally underpinned by two partially distinct neural network: an “intentionality network” involving the rostral area of the mesial frontal cortex, the anterior insula and the inferior parietal lobule and an “self-agency network,” which involves the posterior areas of the mesial frontal cortex (the SMA proper) and the posterior insula.

In the next paragraph, we will discuss the dissociation between the “intentionality network” and the “self-agency network” with reference to their distinctive features.

### How Can the Regions Specifically Associated With Motor Intention and Sense of Self-Agency Be Defined From a Functional Point of View?

Our results are consistent with the view that the mesial frontal areas (pre-SMA and middle cingulum), the anterior insula and the parietal lobes jointly represent a circuit which elaborates motor plans in advance of the action, producing a conscious experience of motor intention. On the contrary, our data suggest that a different set of brain regions, including the SMA proper, the posterior insula, the occipital lobe and the cerebellum, sustain our sense of self-agency.

These networks differ in their distinctive features: the “intentional network” might be conceptualized as a high-level cognitive system, one step or two steps away from detailed action planning or implementation; the “self-agency network” could be seen as a “sensorimotor” system.

In line with these ideas, the pre-SMA belongs to the prefrontal network, rather than to the premotor one (Kim et al., 2010); it is strongly connected with prefrontal cortices and high-level motor areas and it has a specific role in the performance of complex tasks, such as alternation of motor plans, task switching, acquisition of new motor skills and motor selection (Nachev et al., 2007, 2008; De Baene and Brass, 2013). Similarly, the anterior part of the cingulate cortex (BA 24 and 32), also associated with motor intention, has been defined as “the cognitive division” of the cingulate cortex; it can be distinguished from the posterior one based for its cytoarchitecture, patterns of connectivity and functions (Devinsky et al., 1995; Krieghoff et al., 2011): it plays a role in high-level executive functions, such as response selection and conflict monitoring (Bush et al., 2000). Likewise, the anterior insula, given its connections with the frontal and limbic regions (Cauda et al., 2011) and self-referential cognitive (Mayer et al., 2007) or emotional functions (Singer et al., 2009), is regarded as the “cognitive” portion of the insular cortex (Kurth et al., 2010; Cauda et al., 2011).

On the other hand, the SMA proper, associated with the self-agency-network, can be considered as a typical pre-motor area: this region is somatotopically organized and it projects directly to the primary motor cortex (M1) and spinal cord (He et al., 1995), and it is functionally connected with regions related to simple motor control (Kim et al., 2010). The posterior insula, another region linked to the same network, is involved in different sensorimotor processes (Kurth et al., 2010): it is involved in sensory processing (Dupont et al., 2003) and its electrical stimulation in humans produces overt movement elicitation (Showers and Lauer, 1961).

Taken together, evidence from functional data suggests that these two networks may be partially dissociated with respect to both their anatomical and functional features, with “cognitive” motor functions being represented in the intentional network and “executive” motor functions in the agency one (Kim et al., 2010). In the next paragraph, we will discuss this evidence with reference to the different models of action awareness.

### Do the Available Data Permit to Identify a Best Fitting Theory of Action Awareness?

Our findings are directly relevant to the aforementioned cognitive models of action awareness and, more in general, to a theoretical account of motor control. In particular, such complex scenario is partially in contrast with the retrospective framework of action awareness (Wegner and Wheatley, 1999), which considers motor intention and the sense of agency as the result of a common general-domain mechanism. According to this view, we should have observed a unique set of non-motoric brain regions, which are meant to be involved in a general appreciation of causality, shared by intention and agency. Our data show the existence of a set of brain regions associated with both the intention and the agency-specific processes; however, this network is not located in the brain areas typically involved in the high-level processing of causality. Moreover, we provide evidence supporting the existence of two separate networks specifically associated with the two constructs.

Our data fit better with the constructive view of the action awareness experience (Wolpert and Ghahramani, 2000). The comparator model (1) describes motor intention and the sense of agency as distinctive experiences and (2) considers action awareness as an intrinsic property of the action processing that emerges from computational processes similar to those that allow action planning and action-outcome monitoring (Wolpert and Ghahramani, 2000). Our data comply with these arguments, as (1) the motor intention and the sense of agency experiences have been associated with partially-distinct neurofunctional networks and (2) the regions that were associated with the sense of agency are essentially sensorimotor areas. We suggest that action awareness can be regarded as a complex process, which includes almost two distinctive experiences partially dissociable from the neurocognitive point of view: a cognitive process, such as motor intention, underpinned by a high-level cognitive system, and a process more embedded in the sensory-motor system, associated with distinct neurocognitive mechanisms, such as the sense of agency. The strong link between the sense of agency and the sensory-motor system also emerges from the reduced activation of the posterior parietal regions in self-agency conditions, described by the clustering analysis performed on the external-agency dataset^8^. The reduced activation in posterior parietal regions, involved in high-order processes of sensory, multisensory and sensorimotor integration (Berlucchi and Vallar, 2018), is in line with the sensory-attenuation hypothesis put forward by the comparator model: when predictions of the outcome of one’s action make reafferent perception of the actual outcome redundant, the motor system uses the predictions to cancel or attenuate the sensory consequences of the action (Haggard, 2017).

In a nutshell, the involvement of the sensory-motor system in action consciousness implies that, at least for the simplest motor functions that could be addressed in an experimental setting, awareness is neither the prerogative of some kind of general process, hierarchically superimposed on sensory-motor and cognitive functions (see for example Gazzaniga, 1998, “The interpreter”), nor a function that is completely separated from the primary processes that computes the action program.

However, the two cited models might not be considered as mutually exclusive: indeed, components of the two models may play a role in action awareness perhaps in different instances of motor behavior. For example, the retrospective model of awareness could account for action awareness in the context of every-day life events, when the motor plan has been lost due to the temporal distance between action and the need to account for its execution. In this case, we cannot take advantage of the comparison between the predictions and the external feedbacks and we can only rely on a reconstruction of the event authorship and source. However, further studies are needed to support this hypothesis.

## Conclusion

Action awareness is a key feature of our mental life, involving, among other things, motor intentionality and sense of agency.

In this manuscript, we tried to address the hypothesis that the available imaging data could best support one of two popular interpretations on action awareness: the reconstructive model (Wegner and Wheatley, 1999; Wegner, 2003) and the constructive/comparator model (Wolpert, 1997; Wolpert and Ghahramani, 2000).

According to what we found, the second came on top, with many inevitable caveats though: the intrinsic limitations of meta-analyses is one; another caveat comes from the obvious appreciation that any thematic review of additional literature would point to the observation that functional segregations, as those described here, do not imply one-to-one specialization (see the case discussed in Zapparoli et al., 2017, where the intentionality specific networks were also active in conditional action paradigms). Finally, the distributed nature of these networks indicates the need of more behavioral evidence and paradigms that may lead to the assignations of specific roles to the brain areas involved in conscious action awareness. Studies employing functional imaging using causal models, rather than univariate analyses, or non-invasive brain stimulation could be particularly helpful for this purpose.

## Author Contributions

SS, EZ, EP, and LZ reviewed the data for the meta-analyses, performed the analyses, and drafted the manuscript.

## Conflict of Interest Statement

The authors declare that the research was conducted in the absence of any commercial or financial relationships that could be construed as a potential conflict of interest.

## Footnotes

1 https://www.ncbi.nlm.nih.gov/pubmed/

2 At this time, the paper on intentionality by Zapparoli et al. (2018) was not published yet. Accordingly, that data is not included in this meta-analysis. This will be instrumental later on in the paper allowing us to avoid circularity when referring to that paper in our discussion.

3 https://goo.gl/rB2DQx

4 https://www.nitrc.org/projects/mricron

5 www.fil.ion.ucl.ac.uk/spm/software/spm12

6 web.mit.edu/swg/software.htm

7 www.nitrc.org/projects/conn

8 Strictly speaking, the same clusters may imply stronger activity in the external agency conditions. We have no means, at the moment, to support the “signal reduction” hypothesis related to agency in a conclusive manner here.

## Abbreviations

AAL: automatic anatomical labeling
ALE: activation likelihood estimation
CL: cluster
FDR: false discovery rate
fMRI: functional magnetic resonance imaging
FWE: family-wise error
GLM: general linear model
HC: hierarchical clustering
MNI: Montreal Neurological Institute
PET: positron emission tomography
Pre-SMA: pre-supplementary motor area
ROIs: regions of interest
rsFC: resting-state functional connectivity
SMA: supplementary motor area
