# Supplementary material for: The Brain in (Willed) Action: A Meta-Analytical Comparison of Imaging Studies on Motor Intentionality and Sense of Agency
Source: Front Psychol. 2019 Apr 12;10:804. doi: 10.3389/fpsyg.2019.00804 (PMC6473038; doi:10.3389/fpsyg.2019.00804)
Supplement: Supplementary file 1 [file Table_1.docx]

| **Table S1.** Neuroimaging studies included in the meta-analysis on the motor intention (a) and sense of self-agency (b). | | | |  |
| --- | --- | --- | --- | --- |
| **Study** | **Subjects** | **Imaging method** | **Contrast description** | **Adopted statistical threshold** |
| **a. Neuroimaging studies investigating the neurofunctional correlates of motor intention.** | | | | |
| Deiber et al. (1991) | 8 | PET | Intentional conditions vs fixed selection. | p < .05 or p < .01 corrected for multiple comparison |
| Frith et al.  (1991) | 12 | PET | Intention task 1 vs routine task.  Intention task 2 vs routine task. | p < .001 uncorrected |
| Jahanshahi et al. (1995) | 6 | PET | Intentional movements vs. rest.  Intentional vs. externally triggered movements. | p < .05 corrected for multiple comparison |
| Jenkins et al. (2000) | 6 | PET | Intentional finger movements vs rest. | p < .05 corrected for multiple comparison |
| Chaminade et al. (2002) | 9 | PET | Intentional condition vs stimulus-driven condition. | p < .05 corrected for multiple comparison |
| Hunter et al. (2003) | 6 | fMRI | Intentional bottom presses vs rest. | p < .05 corrected for multiple comparison |
| Lau et al. (2004a) | 11 | fMRI | Attention to intention vs. attention to movement (Libet temporal judgment task). | p < .05 corrected for multiple comparison |
| Lau et al. (2004b) | 12 | fMRI | Intentional target choice vs. specified target choice. | p < .05 corrected for multiple comparison |
| Forstmann et al. (2006) | 22 | fMRI | Intentional choice condition vs. forced condition. | p < .001 uncorrected |
| van Eimeren et al. (2006) | 12 | fMRI | Intentional response selection vs. no response selection. | p < .05 FDR corrected for multiple comparison |
| Mueller et al. (2007) | 15 | fMRI | Intentionally vs. externally selected actions. | p < .001 corrected for multiple comparison |
| Krieghoff et al. (2009) | 14 | fMRI | Intentional action selection vs. external action selection (time of decision and time of implementation of decision).  Intentional action timing vs. external action timing. | p < .001 corrected for multiple comparison |
| Kuhn and Brass (2009) | 17 | fMRI | Intentional decision for action vs. inhibition of action.  Intentional decision against action vs. inhibition of action. | p < .001 uncorrected |
| Hoffstaedter et al. (2013) | 35 | fMRI | Intentional choice vs no choice condition.  Intentional choice vs. timed condition. | p < .05 FWE-corrected, cluster level |
| Schel et al. (2014) | 24 | fMRI | Intentional action vs. stimulus-driven action. | p < .05 FDR-corrected |
| Lynn et al. (2016) | 20 | fMRI | Intentional choice action vs forced action. | p < .05 corrected for multiple comparison |
| Wisniewski et al. (2016) | 31 | fMRI | Intentional choice vs. cued choice. | p < .05 FWE-corrected, voxel level |
| **b. Neuroimaging studies investigated the functional correlates of sense of self-agency.** | | | | |
| Farrer and Frith (2002) | 12 | fMRI | Self-agency vs. control condition.  Self-agency vs. external-agency. | p < .001 uncorrected |
| Farrer et al. (2003) | 8 | PET | Parametric function of visuo-motor congruency degree (congruency = self-agency; no congruency = external-agency). | p < .0001 uncorrected |
| Leube et al. (2003a) | 18 | fMRI | Negative correlation with visuo-motor congruency degree (congruency = self-agency; no congruency = external-agency). | p < .05 corrected for multiple comparison |
| Leube et al. (2003b) | 6 | fMRI | Visual feedback of own actions (self-agency) vs. visual feedback of external’s actions (external-agency). | p < .05 corrected for multiple comparison |
| Matsuzawa et al. (2005) | 6 | fMRI | Visuo-motor congruency (self-agency) vs. rest. | p < .001 corrected for multiple comparison |
| Schnell et al. (2007) | 15 | fMRI | Visuo-motor congruency (self-agency) vs. visuo-motor discrepancy (external-agency). | p < .01 FDR-corrected |
| Kontaris et al. (2009) | 11 | fMRI | Compatible (self-agency) vs. incompatible visual feedback (external-agency). | p < .005 corrected for multiple comparison |
| Spengler et al. (2009) | 18 | fMRI | Parametric function of visuo-motor congruency degree (self-agency). | p < .05 corrected for multiple comparison |
| Tsakiris et al. (2010) | 19 | fMRI | Movement with synchronous feedback (self-agency) vs. movement with asynchronous feedback (external-agency). | p < .001 uncorrected |
| Chambon et al. (2013) | 22 | fMRI | Compatible action priming (self-agency) vs. not compatible action priming (external-agency). | p < .05 FDR-corrected |
| Fukushima et al. (2013) | 17 | fMRI | Judgment of self-agency vs judgment of external-agency. | p < .001 uncorrected |
| Kuhn et al. (2013) | 17 | fMRI | Parametric function of temporal compression (intentional binding paradigm for self vs external-agency). | p < .001 uncorrected |
| Renes et al. (2015) | 21 | fMRI | Self-agency vs. external-agency. | P < .05 FWE-corrected, cluster level |
| de Bézenac et al. (2016) | 24 | fMRI | Main effect of self-agency condition. | P < .05 corrected for multiple comparison |

**References**

Chambon, V., Wenke, D., Fleming, S.M., Prinz, W., Haggard, P. (2013) An online neural substrate for sense of agency. Cereb Cortex, 23:1031-7.

Chaminade, T., Decety, J. (2002) Leader or follower? Involvement of the inferior parietal lobule in agency. Neuroreport, 13:1975-8.

de Bézenac, C.E., Sluming, V., Gouws, A., Corcoran, R. (2016) Neural response to modulating the probability that actions of self or other result in auditory tones: A parametric fMRI study into causal ambiguity. Biol Psychol, 119:64-78.

Deiber, M.P., Passingham, R.E., Colebatch, J.G., Friston, K.J., Nixon, P.D., Frackowiak, R.S. (1991) Cortical areas and the selection of movement: a study with positron emission tomography. Exp Brain Res, 84:393-402.

Farrer, C., Franck, N., Georgieff, N., Frith, C.D., Decety, J., Jeannerod, M. (2003) Modulating the experience of agency: a positron emission tomography study. Neuroimage, 18:324-33.

Farrer, C., Frith, C.D. (2002) Experiencing oneself vs another person as being the cause of an action: the neural correlates of the experience of agency. Neuroimage, 15:596-603.

Forstmann, B.U., Brass, M., Koch, I., von Cramon, D.Y. (2006) Voluntary selection of task sets revealed by functional magnetic resonance imaging. J Cogn Neurosci, 18:388-98.

Frith, C.D., Friston, K., Liddle, P.F., Frackowiak, R.S. (1991) Willed action and the prefrontal cortex in man: a study with PET. Proc Biol Sci, 244:241-6.

Fukushima, H., Goto, Y., Maeda, T., Kato, M., Umeda, S. (2013) Neural substrates for judgment of self-agency in ambiguous situations. PLoS One, 8:e72267.

Hoffstaedter, F., Grefkes, C., Zilles, K., Eickhoff, S.B. (2013) The "what" and "when" of self-initiated movements. Cereb Cortex, 23:520-30.

Hunter, M.D., Farrow, T.F., Papadakis, N.G., Wilkinson, I.D., Woodruff, P.W., Spence, S.A. (2003) Approaching an ecologically valid functional anatomy of spontaneous "willed" action. Neuroimage, 20:1264-9.

Jahanshahi, M., Jenkins, I.H., Brown, R.G., Marsden, C.D., Passingham, R.E., Brooks, D.J. (1995) Self-initiated versus externally triggered movements. I. An investigation using measurement of regional cerebral blood flow with PET and movement-related potentials in normal and Parkinson's disease subjects. Brain, 118 ( Pt 4):913-33.

Jenkins, I.H., Jahanshahi, M., Jueptner, M., Passingham, R.E., Brooks, D.J. (2000) Self-initiated versus externally triggered movements. II. The effect of movement predictability on regional cerebral blood flow. Brain, 123 ( Pt 6):1216-28.

Kontaris, I., Wiggett, A.J., Downing, P.E. (2009) Dissociation of extrastriate body and biological-motion selective areas by manipulation of visual-motor congruency. Neuropsychologia, 47:3118-24.

Krieghoff, V., Brass, M., Prinz, W., Waszak, F. (2009) Dissociating what and when of intentional actions. Front Hum Neurosci, 3:3.

Kühn, S., Brass, M. (2009) When doing nothing is an option: the neural correlates of deciding whether to act or not. Neuroimage, 46:1187-93.

Kühn, S., Brass, M., Haggard, P. (2013) Feeling in control: Neural correlates of experience of agency. Cortex, 49:1935-42.

Lau, H.C., Rogers, R.D., Haggard, P., Passingham, R.E. (2004a) Attention to intention. Science, 303:1208-10.

Lau, H.C., Rogers, R.D., Ramnani, N., Passingham, R.E. (2004b) Willed action and attention to the selection of action. Neuroimage, 21:1407-15.

Leube, D.T., Knoblich, G., Erb, M., Grodd, W., Bartels, M., Kircher, T.T. (2003a) The neural correlates of perceiving one's own movements. Neuroimage, 20:2084-90.

Leube, D.T., Knoblich, G., Erb, M., Kircher, T.T. (2003b) Observing one's hand become anarchic: an fMRI study of action identification. Conscious Cogn, 12:597-608.

Lynn, M.T., Demanet, J., Krebs, R.M., Van Dessel, P., Brass, M. (2016) Voluntary inhibition of pain avoidance behavior: an fMRI study. Brain Struct Funct, 221:1309-20.

Matsuzawa, M., Matsuo, K., Sugio, T., Kato, C., Nakai, T. (2005) Temporal relationship between action and visual outcome modulates brain activation: an fMRI study. Magn Reson Med Sci, 4:115-21.

Mueller, V.A., Brass, M., Waszak, F., Prinz, W. (2007) The role of the preSMA and the rostral cingulate zone in internally selected actions. Neuroimage, 37:1354-61.

Renes, R.A., van Haren, N.E., Aarts, H., Vink, M. (2015) An exploratory fMRI study into inferences of self-agency. Soc Cogn Affect Neurosci, 10:708-12.

Schel, M.A., Kühn, S., Brass, M., Haggard, P., Ridderinkhof, K.R., Crone, E.A. (2014) Neural correlates of intentional and stimulus-driven inhibition: a comparison. Front Hum Neurosci, 8:27.

Schnell, K., Heekeren, K., Schnitker, R., Daumann, J., Weber, J., Hesselmann, V., Möller-Hartmann, W., Thron, A., Gouzoulis-Mayfrank, E. (2007) An fMRI approach to particularize the frontoparietal network for visuomotor action monitoring: Detection of incongruence between test subjects' actions and resulting perceptions. Neuroimage, 34:332-41.

Spengler, S., von Cramon, D.Y., Brass, M. (2009) Was it me or was it you? How the sense of agency originates from ideomotor learning revealed by fMRI. Neuroimage, 46:290-8.

Tsakiris, M., Longo, M.R., Haggard, P. (2010) Having a body versus moving your body: neural signatures of agency and body-ownership. Neuropsychologia, 48:2740-9.

van Eimeren, T., Wolbers, T., Münchau, A., Büchel, C., Weiller, C., Siebner, H.R. (2006) Implementation of visuospatial cues in response selection. Neuroimage, 29:286-94.

Wisniewski, D., Goschke, T., Haynes, J.D. (2016) Similar coding of freely chosen and externally cued intentions in a fronto-parietal network. Neuroimage, 134:450-458.
