# Supplementary material for: The Brain in (Willed) Action: A Meta-Analytical Comparison of Imaging Studies on Motor Intentionality and Sense of Agency
Source: Front Psychol. 2019 Apr 12;10:804. doi: 10.3389/fpsyg.2019.00804 (PMC6473038; doi:10.3389/fpsyg.2019.00804)
Supplement: Supplementary file 2 [file Table_2.docx]

**Table S2.** Neuroimaging studies included in the meta-analysis on the sense of external-agency.

| **Study** | **Subjects** | **Imaging method** | **Contrast description** | **Adopted statistical threshold** |
| --- | --- | --- | --- | --- |
| Farrer and Frith (2002) | 12 | fMRI | External-agency vs control condition; external-agency vs self-agency. | p < .001 uncorrected |
| Farrer et al. (2003) | 8 | PET | Parametric function of visuo-motor congruency degree (no congruency = external-agency; congruency = self-agency). | p < .05 corrected for multiple comparison or p < .0001 uncorrected |
| Leube et al. (2003) | 18 | fMRI | Positive correlation with visuo-motor congruency degree (external-agency). | p < .05 corrected for multiple comparison |
| Matsuzawa et al. (2005) | 6 | fMRI | Visuo-motor incongruency (external-agency) vs. rest. | p < .001 corrected for multiple comparison |
| Balslev et al. (2006) | 16 | fMRI | Visuo-motor incongruency (external-agency) vs. visuo-motor congruency (self-agency). | p < .001 corrected for multiple comparison |
| Schnell et al. (2007) | 15 | fMRI | Visuo-motor incongruency (external-agency) vs. visuo-motor congruency (self-agency). | p < .01 FDR-corrected |
| Farrer et al. (2008) | 18 | fMRI | Main effect of external-agency condition. | p < .001 uncorrected |
| Spengler et al. (2009) | 18 | fMRI | Parametric function of visuo-motor incongruency degree. | p < .05 corrected for multiple comparison |
| Kontaris et al. (2009) | 11 | fMRI | Visuo-motor incongruency (external-agency) vs. visuo-motor congruency (self-agency). | p < .005 corrected for multiple comparison |
| Nahab et al. (2011) | 20 | fMRI | Positive correlation with external-agency condition. | p < .05 corrected for multiple comparison, cluster level |
| van Kemenade et al. (2017) | 20 | fMRI | Positive correlation with visuo-motor congruency degree (external-agency). | p < .001 uncorrected or p < .05 FWE-corrected, cluster level |

**References**

Balslev, D., Nielsen, F.A., Lund, T.E., Law, I., Paulson, O.B. (2006) Similar brain networks for detecting visuo-motor and visuo-proprioceptive synchrony. Neuroimage, 31:308-12.

Farrer, C., Franck, N., Georgieff, N., Frith, C.D., Decety, J., Jeannerod, M. (2003) Modulating the experience of agency: a positron emission tomography study. Neuroimage, 18:324-33.

Farrer, C., Frey, S.H., Van Horn, J.D., Tunik, E., Turk, D., Inati, S., Grafton, S.T. (2008) The angular gyrus computes action awareness representations. Cereb Cortex, 18:254-61.

Farrer, C., Frith, C.D. (2002) Experiencing oneself vs another person as being the cause of an action: the neural correlates of the experience of agency. Neuroimage, 15:596-603.

Kontaris, I., Wiggett, A.J., Downing, P.E. (2009) Dissociation of extrastriate body and biological-motion selective areas by manipulation of visual-motor congruency. Neuropsychologia, 47:3118-24.

Leube, D.T., Knoblich, G., Erb, M., Grodd, W., Bartels, M., Kircher, T.T. (2003) The neural correlates of perceiving one's own movements. Neuroimage, 20:2084-90.

Matsuzawa, M., Matsuo, K., Sugio, T., Kato, C., Nakai, T. (2005) Temporal relationship between action and visual outcome modulates brain activation: an fMRI study. Magn Reson Med Sci, 4:115-21.

Nahab, F.B., Kundu, P., Gallea, C., Kakareka, J., Pursley, R., Pohida, T., Miletta, N., Friedman, J., Hallett, M. (2011) The neural processes underlying self-agency. Cereb Cortex, 21:48-55.

Schnell, K., Heekeren, K., Schnitker, R., Daumann, J., Weber, J., Hesselmann, V., Möller-Hartmann, W., Thron, A., Gouzoulis-Mayfrank, E. (2007) An fMRI approach to particularize the frontoparietal network for visuomotor action monitoring: Detection of incongruence between test subjects' actions and resulting perceptions. Neuroimage, 34:332-41.

Spengler, S., von Cramon, D.Y., Brass, M. (2009) Was it me or was it you? How the sense of agency originates from ideomotor learning revealed by fMRI. Neuroimage, 46:290-8.

van Kemenade, B.M., Arikan, B.E., Kircher, T., Straube, B. (2017) The angular gyrus is a supramodal comparator area in action-outcome monitoring. Brain Struct Funct.
