# Supplementary material for: The Brain in (Willed) Action: A Meta-Analytical Comparison of Imaging Studies on Motor Intentionality and Sense of Agency
Source: Front Psychol. 2019 Apr 12;10:804. doi: 10.3389/fpsyg.2019.00804 (PMC6473038; doi:10.3389/fpsyg.2019.00804)
Supplement: Supplementary file 3 [file Table_3.docx]

| **Table S3.** Cluster analysis results: motor intention and sense of self-agency. For each cluster we report: the cluster ID, the number of foci included in the cluster (k), the centroid coordinates in the MNI stereotaxic space and the standard deviation along the three axes (x.y.z), the p values associated with the binomial test. The clusters significantly associated with either factor are highlighted in bold. | | | | | | | | | | | | | | | | | | | | |
| --- | --- | --- | --- | --- | --- | --- | --- | --- | --- | --- | --- | --- | --- | --- | --- | --- | --- | --- | --- | --- |
| ***Brain regions (BA)*** | **MNI coordinates** | | | | | | | | | | | | | | | | | | | |
|  | **Left hemisphere** | | | | | | | | | | **Right hemisphere** | | | | | | | | | |
|  | ***Cluster ID*** | ***k*** | **MNI coordinates** | | | **Standard deviation** | | | **P values** | | ***Cluster ID*** | ***k*** | **MNI coordinates** | | | **Standard deviation** | | | **P values** | |
|  |  |  | ***x*** | ***y*** | ***z*** | ***x*** | ***y*** | ***z*** | **Intention** | **Agency** |  |  | ***x*** | ***y*** | ***z*** | ***x*** | ***y*** | ***z*** | **Intention** | **Agency** |
| Inferior frontal gyrus pars orbitalis (10) | 40 | 3 | -29 | 29 | -10 | 5.7 | 2.6 | 9.1 | 0.81 | 0.63 | 61 | 2 | 3 | 65 | -7 | 7.5 | 1.2 | 15.4 | 1 | 0.08 |
| Inferior frontal gyrus pars triangularis (45) | 6 | 5 | -43 | 32 | 22 | 2.2 | 4.1 | 8.1 | 0.19 | 1 |  |  |  |  |  |  |  |  |  |  |
| Inferior frontal gyrus pars triangularis (47) |  |  |  |  |  |  |  |  |  |  | 57 | 6 | 41 | 25 | 5 | 7.5 | 4.6 | 9.4 | 0.46 | 0.86 |
| Inferior frontal gyrus pars opercularis | 18 | 4 | -55 | 12 | 1 | 5.3 | 2.8 | 3.7 | 0.68 | 0.74 | 54 | 5 | 51 | 7 | 18 | 3.7 | 1.3 | 3.4 | 0.56 | 0.81 |
|  | 1 | 3 | -45 | -25 | 21 | 2.3 | 7 | 4 | 0.98 | 0.19 | 10 | 3 | 53 | -10 | 12 | 10.8 | 3 | 3.9 | 0.81 | 0.63 |
| Middle frontal gyrus (46) | 49 | 5 | -37 | 53 | 10 | 2.7 | 6.7 | 4.9 | 0.19 | 1 | 51 | 6 | 31 | 48 | 24 | 7.8 | 5.2 | 5 | 0.46 | 0.86 |
|  | 5 | 7 | -36 | 41 | 27 | 4.4 | 2.7 | 3.3 | 0.37 | 0.9 |  |  |  |  |  |  |  |  |  |  |
| Middle frontal gyrus (45) |  |  |  |  |  |  |  |  |  |  | 68 | 7 | 43 | 34 | 30 | 4.4 | 8.1 | 4 | 0.37 | 0.9 |
| Middle frontal gyrus (10) |  |  |  |  |  |  |  |  |  |  | 50 | 5 | 33 | 54 | 4 | 4.9 | 3.9 | 8.4 | 0.19 | 1 |
| Middle frontal gyrus (9) | 60 | 3 | -41 | 14 | 44 | 4.7 | 5.9 | 3.8 | 0.98 | 0.19 |  |  |  |  |  |  |  |  |  |  |
| Superior frontal gyrus (6) | 13 | 4 | -21 | 0 | 67 | 3.1 | 3.5 | 3.2 | 0.68 | 0.74 |  |  |  |  |  |  |  |  |  |  |
|  | 14 | 4 | -23 | -3 | 52 | 3 | 7.2 | 5.1 | 0.26 | 1 |  |  |  |  |  |  |  |  |  |  |
| Superior frontal gyrus (9) | 48 | 2 | -21 | 56 | 32 | 1.4 | 5.7 | 11.3 | 0.92 | 0.49 | 35 | 1 | 20 | 36 | 52 | 0 | 0 | 0 | 1 | 0.28 |
|  |  |  |  |  |  |  |  |  |  |  | 66 | 6 | 25 | 8 | 58 | 6.7 | 5.9 | 4.6 | 0.46 | 0.86 |
| Anterior cingulum (32) |  |  |  |  |  |  |  |  |  |  | 36 | 8 | 4 | 31 | 31 | 5.2 | 5.5 | 3.3 | 0.29 | 0.93 |
| **Middle cingulum (24)** |  |  |  |  |  |  |  |  |  |  | **53** | **17** | **1** | **19** | **39** | **4.6** | **4** | **3.4** | **0.03** | **1** |
| Middle cingulum (23) | 52 | 2 | -2 | -17 | 48 | 2.8 | 1.4 | 5.7 | 1 | 0.08 | 3 | 3 | 0 | -30 | 27 | 0.9 | 5.5 | 4.9 | 0.81 | 0.63 |
| Posterior cingulum (30) |  |  |  |  |  |  |  |  |  |  | 4 | 3 | 3 | -47 | 23 | 6.5 | 4 | 3.6 | 0.81 | 0.63 |
| **Pre-supplementary motor area (6)** | **58** | **10** | **-3** | **12** | **53** | **7.1** | **6.5** | **2.5** | **0.04** | **1** |  |  |  |  |  |  |  |  |  |  |
| **Supplementary motor area (6)** | **24** | **5** | **-7** | **-4** | **69** | **4.7** | **5** | **5** | **1** | **0.02** | 23 | 5 | 4 | 6 | 67 | 2.3 | 4.2 | 2.7 | 0.56 | 0.81 |
| Precentral gyrus (6) | 20 | 5 | -39 | -12 | 58 | 6.8 | 3.2 | 8.4 | 0.98 | 0.14 |  |  |  |  |  |  |  |  |  |  |
|  | 17 | 2 | -58 | 5 | 18 | 2.2 | 1 | 3.3 | 1 | 0.08 |  |  |  |  |  |  |  |  |  |  |
| Precentral gyrus (4) | 19 | 3 | -33 | -26 | 58 | 3.9 | 6 | 5.8 | 0.37 | 1 |  |  |  |  |  |  |  |  |  |  |
| Postcentral gyrus (3) |  |  |  |  |  |  |  |  |  |  | 42 | 3 | 38 | -26 | 52 | 0.6 | 2.1 | 5.4 | 1 | 0.02 |
| **Anterior insula** | **41** | **9** | **-41** | **17** | **-2** | **4.9** | **4.4** | **5** | **0.05** | **1** | 31 | 7 | 43 | 9 | 0 | 4.3 | 1.8 | 4.5 | 0.69 | 0.63 |
| **Posterior insula** | **28** | **7** | **-41** | **2** | **1** | **4.4** | **4.1** | **5.9** | **1** | **0.02** |  |  |  |  |  |  |  |  |  |  |
|  | 2 | 2 | -39 | -21 | 3 | 13.1 | 2 | 0.7 | 0.92 | 0.49 |  |  |  |  |  |  |  |  |  |  |
| Superior parietal gyrus (2) |  |  |  |  |  |  |  |  |  |  | 25 | 2 | 26 | -46 | 59 | 2.1 | 7.8 | 6.4 | 1 | 0.08 |
| **Superior parietal gyrus (7)** | 63 | 4 | -17 | -63 | 58 | 3.5 | 6.5 | 2.9 | 0.26 | 1 | **45** | **9** | **18** | **-67** | **52** | **5.4** | **3.6** | **4.1** | **0.05** | **1** |
| **Inferior parietal lobule (40)** | 47 | 10 | -49 | -43 | 53 | 3.6 | 6.9 | 2.9 | 0.18 | 0.96 | **22** | **13** | **39** | **-45** | **41** | **4.4** | **5.3** | **3.8** | **0.01** | **1** |
|  | 8 | 5 | -40 | -40 | 40 | 3.7 | 3.4 | 2.8 | 0.19 | 1 | 26 | 7 | 43 | -49 | 54 | 3.2 | 7.7 | 3.6 | 0.37 | 0.9 |
| Supramarginal gyrus (40) | 7 | 3 | -55 | -39 | 35 | 3.4 | 2.4 | 3.9 | 0.37 | 1 | 43 | 7 | 55 | -34 | 45 | 2.9 | 6.3 | 5.6 | 0.1 | 1 |
| Angular gyrus (39) |  |  |  |  |  |  |  |  |  |  | 21 | 2 | 53 | -53 | 37 | 2.8 | 4.2 | 1.4 | 0.51 | 1 |
| Superior temporal gyrus | 44 | 2 | -67 | -18 | 11 | 3.1 | 10.4 | 5.3 | 0.92 | 0.49 |  |  |  |  |  |  |  |  |  |  |
| Superior temporal pole (38) |  |  |  |  |  |  |  |  |  |  | 32 | 4 | 58 | 10 | -3 | 3.4 | 3.7 | 6.9 | 0.99 | 0.07 |
|  |  |  |  |  |  |  |  |  |  |  | 37 | 2 | 27 | 9 | -18 | 4.3 | 4.2 | 5.2 | 1 | 0.08 |
| Superior temporal gyrus (22) | 9 | 2 | 58 | -29 | 10 | 2.5 | 4.2 | 11.3 | 0.92 | 0.49 |  |  |  |  |  |  |  |  |  |  |
| Precuneus (7) |  |  |  |  |  |  |  |  |  |  | 46 | 6 | 3 | -64 | 47 | 4.4 | 6.5 | 3.9 | 0.46 | 0.86 |
| Fusiform gyrus (19) |  |  |  |  |  |  |  |  |  |  | 16 | 3 | 36 | -66 | -15 | 5.2 | 5.2 | 5.2 | 0.81 | 0.63 |
| Superior occipital gyrus (7) |  |  |  |  |  |  |  |  |  |  | 12 | 3 | 28 | -70 | 38 | 6 | 3.6 | 3 | 0.81 | 0.63 |
| Middle occipital gyrus (19) | 33 | 2 | -29 | -83 | 23 | 2.1 | 2.1 | 2.1 | 0.92 | 0.49 | 11 | 3 | 33 | -78 | 23 | 4.9 | 6 | 2 | 0.98 | 0.19 |
| Middle occipital gyrus | 34 | 3 | -40 | -63 | 26 | 10.4 | 4.2 | 6 | 0.98 | 0.19 |  |  |  |  |  |  |  |  |  |  |
| **Calcarine scissure (18)** | 39 | 1 | -15 | -56 | 8 | 0 | 0 | 0 | 1 | 0.28 | **59** | **5** | **18** | **-90** | **-1** | **6** | **4.2** | **8.4** | **1** | **0.02** |
| Inferior occipital gyrus (19) | 55 | 2 | -35 | -86 | -5 | 7.1 | 11.3 | 4.2 | 1 | 0.08 | 15 | 3 | 39 | -82 | -9 | 1 | 8.2 | 3.1 | 1 | 0.02 |
| Cerebellum crus | 56 | 3 | -11 | -73 | -27 | 11.5 | 9.7 | 5.5 | 1 | 0.02 |  |  |  |  |  |  |  |  |  |  |
| **Cerebellum** | 62 | 11 | -31 | -57 | -32 | 4.3 | 2.2 | 4.7 | 0.36 | 0.86 | **67** | **5** | **24** | **-53** | **-27** | **8.8** | **7.8** | **6.7** | **1** | **0.02** |
| Thalamus | 65 | 7 | -11 | -16 | 8 | 4 | 6.3 | 6.8 | 0.69 | 0.63 | 30 | 3 | 12 | -12 | 12 | 4.7 | 7.2 | 8 | 0.98 | 0.19 |
| Putamen | 64 | 8 | -16 | 8 | -4 | 2.2 | 4.8 | 2.5 | 0.29 | 0.93 | 29 | 2 | 32 | -11 | 2 | 2.5 | 4.7 | 11.3 | 1 | 0.08 |
|  | 27 | 4 | -27 | -1 | 0 | 1.4 | 2.8 | 2.8 | 0.93 | 0.32 |  |  |  |  |  |  |  |  |  |  |
| Pallidum |  |  |  |  |  |  |  |  |  |  | 38 | 11 | 18 | 7 | -1 | 4.6 | 3.9 | 3.8 | 0.14 | 0.97 |
|  | | | | | | | | | | | | | | | | | | | | |
