# Supplementary material for: The Brain in (Willed) Action: A Meta-Analytical Comparison of Imaging Studies on Motor Intentionality and Sense of Agency
Source: Front Psychol. 2019 Apr 12;10:804. doi: 10.3389/fpsyg.2019.00804 (PMC6473038; doi:10.3389/fpsyg.2019.00804)
Supplement: Supplementary file 4 [file Table_4.docx]

**Table S4.** Functional Connectivity results. **a)** Brain regions specifically functionally connected with the **pre-SMA** (intentionality network); **b)** Brain areas specifically functionally connected with the **SMA** (self-agency network); **c)** Conjunction analysis between **pre-SMA** (intentionality network) and **SMA** networks (self-agency network). * FWE .05 correction (voxel level). ° FWE .05 correction (cluster level).

| **Brain regions (BA)** | **MNI coordinates** | | | | | | | | | |
| --- | --- | --- | --- | --- | --- | --- | --- | --- | --- | --- |
|  |  |  |  |  |  |  |  |  |  |  |
|  | **Left hemisphere** | | | | | **Right hemisphere** | | | | |
|  | **x** | **y** | **z** | **Z-score** | **P-value** | **x** | **y** | **z** | **Z-score** | **P-value** |
| **a. pre-SMA > SMA** | | | | | | | | | | |
| Inferior frontal gyrus pars orbitalis (47) | -36 | 24 | -8 | 4.52° | 0.000003 |  |  |  |  |  |
|  | -36 | 28 | -6 | 4.36° | 0.000006 |  |  |  |  |  |
|  | -36 | 32 | -4 | 4.31° | 0.000008 |  |  |  |  |  |
|  | -36 | 34 | 0 | 3.94° | 0.00004 |  |  |  |  |  |
|  | -40 | 32 | 2 | 3.72° | 0.0001 |  |  |  |  |  |
|  | -26 | 26 | -6 | 3.59° | 0.0002 |  |  |  |  |  |
| Inferior frontal gyrus pars triangularis (45) | -40 | 30 | 6 | 3.29° | 0.0005 |  |  |  |  |  |
| Inferior frontal gyrus pars triangularis | -38 | 18 | 20 | 3.80° | 0.00007 |  |  |  |  |  |
|  | -56 | 18 | 6 | 3.64° | 0.0001 |  |  |  |  |  |
| Inferior frontal gyrus pars opercularis (44) | -50 | 8 | 26 | 4.87°* | 0.0000006 |  |  |  |  |  |
|  | -52 | 12 | 20 | 4.38° | 0.000006 |  |  |  |  |  |
| Inferior frontal gyrus pars opercularis | -48 | 18 | 18 | 3.88° | 0.00005 |  |  |  |  |  |
|  | -52 | 14 | 10 | 3.66° | 0.0001 |  |  |  |  |  |
| Anterior cingulum (32) |  |  |  |  |  | 12 | 30 | 22 | 4.67° | 0.000002 |
|  |  |  |  |  |  | 10 | 28 | 30 | 4.24° | 0.00001 |
|  |  |  |  |  |  | 18 | 36 | 20 | 3.64° | 0.0001 |
| Anterior cingulum (24) | -4 | 28 | 24 | 3.69° | 0.0001 |  |  |  |  |  |
|  | -2 | 30 | 28 | 3.57° | 0.0002 |  |  |  |  |  |
| Precentral gyrus (6) | -46 | -2 | 52 | 3.27° | 0.0005 |  |  |  |  |  |
|  | -32 | 4 | 48 | 5.24°* | 8.029E-07 |  |  |  |  |  |
|  | -36 | 0 | 46 | 4.78° | 8.765E-07 |  |  |  |  |  |
|  | -44 | 2 | 60 | 3.46° | 0.0003 |  |  |  |  |  |
| Anterior insula | -34 | 2I0 | -6 | 4.49° | 0.000004 |  |  |  |  |  |
| **b. SMA > pre-SMA** | | | | | | | | | | |
| Middle cingulum | -2 | -6 | 52 | 3.37° | 0.0004 |  |  |  |  |  |
| Precentral gyrus (6) |  |  |  |  |  | 24 | -24 | 72 | 3.66° | 0.0001 |
| Paracentral lobule (4) | -14 | -30 | 76 | 3.37° | 0.0004 | 18 | -22 | 72 | 3.40° | 0.0003 |
|  | -16 | -18 | 70 | 3.41° | 0.0003 |  |  |  |  |  |
| Postcentral gyrus (3) | -22 | -30 | 68 | 4.08° | 0.00002 | 28 | -28 | 56 | 3.29° | 0.0005 |
|  | -20 | -28 | 58 | 3.96° | 0.00004 | 24 | -30 | 58 | 3.19° | 0.0007 |
|  | -18 | -30 | 62 | 3.75° | 0.00009 | 24 | -34 | 64 | 3.62° | 0.0001 |
|  |  |  |  |  |  | 24 | -38 | 66 | 3.57° | 0.0003 |
|  |  |  |  |  |  | 24 | -28 | 50 | 3.52° | 0.0002 |
|  |  |  |  |  |  | 24 | -26 | 54 | 3.44° | 0.0003 |
| Postcentral gyrus (2) |  |  |  |  |  | 28 | -42 | 66 | 3.47° | 0.0003 |
|  | -10 | -32 | 76 | 3.31° | 0.0005 |  |  |  |  |  |
| Superior parietal lobule (2) |  |  |  |  |  | 20 | -46 | 66 | 4.09° | 0.00002 |
| **c. Conjunction between pre-SMA and SMA networks** | | | | | | | | | | |
| Inferior frontal gyrus pars opercularis | -50 | 2 | 2 | 5.61°* | 0.00000001 |  |  |  |  |  |
| Middle frontal gyrus (6) | -28 | -8 | 52 | 5.25°* | 0.00000008 |  |  |  |  |  |
| Anterior cingulum (24) | -6 | 18 | 30 | 5.21°* | 0.00000009 |  |  |  |  |  |
| Middle cingulum (24) | -4 | 4 | 46 | 6.76°* | 0.000000000007 |  |  |  |  |  |
|  | -6 | 10 | 36 | 5.66°* | 0.000000008 |  |  |  |  |  |
| Middle cingulum (32) |  |  |  |  |  | 8 | 10 | 40 | 6.21°* | 0.0000000003 |
| Supplementary motor area (6) | -6 | 4 | 64 | 7.81°* | 0.000000000000003 | 8 | 4 | 64 | 6.95°* | 0.000000000002 |
|  | -2 | 2 | 64 | 7.67°* | 0.000000000000009 |  |  |  |  |  |
| Supplementary motor area |  |  |  |  |  | 0 | 2 | 50 | 6.95°* | 0.000000000002 |
|  |  |  |  |  |  | 0 | 0 | 54 | 6.88°* | 0.000000000003 |
| Anterior insula | -32 | 14 | 4 | 5.43°* | 0.00000003 | 34 | 18 | 2 | 4.97°* | 0.0000003 |
| Middle insula | -46 | 8 | -4 | 5.31°* | 0.00000005 | 40 | 6 | 2 | 5.26°* | 0.00000007 |
|  | -34 | 8 | 6 | 5.06°* | 0.0000002096 | 46 | 8 | 6 | 5.14°* | 0.0000001 |
|  |  |  |  |  |  | 42 | 8 | -6 | 5.1°* | 0.0000002 |
| Posterior insula | -44 | 2 | 8 | 5.28°* | 0.00000006 | 40 | 4 | 8 | 5.13°* | 0.0000001 |
| Supramarginal gyrus | -56 | -32 | 26 | 4.85°* | 0.0000006 |  |  |  |  |  |
| Superior temporal pole | -50 | 10 | -6 | 5.2°* | 0.0000001 | 54 | 8 | 0 | 5.41°* | 0.00000003 |
| Putamen | -32 | 6 | -2 | 5.49°* | 0.00000002 |  |  |  |  |  |
|  | -30 | 8 | 2 | 5.36°* | 0.00000004 |  |  |  |  |  |
